# The Efficacy of Beta-Blockers in Patients With Long QT Syndrome 1–3 According to Individuals’ Gender, Age, and QTc Intervals: A Network Meta-analysis

**DOI:** 10.3389/fphar.2020.579525

**Published:** 2020-12-14

**Authors:** Lu Han, Fuxiang Liu, Qing Li, Tao Qing, Zhenyu Zhai, Zirong Xia, Juxiang Li

**Affiliations:** Department of Cardiovascular Medicine, The Second Affiliated Hospital of Nanchang University, Nanchang, China

**Keywords:** beta-blockers, atenolol, propranolol, metoprolol, nadolol, cardiac events

## Abstract

Long QT syndrome (LQTS) is an arrhythmic heart disease caused by congenital genetic mutations, and results in increased occurrence rates of polymorphic ventricular tachyarrhythmias and sudden cardiac death (SCD). Clinical evidence from numerous previous studies suggested that beta blockers (BBs), including atenolol, propranolol, metoprolol, and nadolol, exhibit different efficacies for reducing the risk of cardiac events (CEs), such as syncope, arrest cardiac arrest (ACA), and SCD, in patients with LQTS. In this study, we identified relevant studies in MEDLINE, PubMed, embase, and Cochrane databases and performed a meta-analysis to assess the relationship between the rate of CEs and LQTS individuals with confounding variables, including different gender, age, and QTc intervals. Moreover, a network meta-analysis was not only established to evaluate the effectiveness of different BBs, but also to provide the ranked efficacies of BBs treatment for preventing the recurrence of CEs in LQT1 and LQT2 patients. In conclusion, nadolol was recommended as a relatively effective strategy for LQT2 in order to improve the prognosis of patients during a long follow-up period.

## Introduction

Congenital long QT syndrome (LQTS) is characterized by a prolonged QT interval and action potential duration (APD). Patients with LQTS have a propensity to develop ventricular tachycardia (VT) and also have a higher rate of cardiac events (CEs) (Arking et al., 2014). The three major genotypes of LQTS, LQT1, LQT2, and LQT3, account for 80–90% of all 15 gene-mutations identified in LQTS patients (Tester and Ackerman, 2014). LQT1, as the prevailing inherited genotype of LQTS, results from gain-of-function mutations in a slow potassium (K^+^) outward current channel encoded by KCNQ1. LQT2 is associated with dysfunction of a rapid K^+^ channel encoded by the KCNH2 gene. Mutations in the SCN5A gene trigger enhanced levels of late sodium (Na^+^) inward current, which is the pathomechanism of LQT3 (Maguy et al., 2020).

Although there are a few novel targeted treatments for LQTS, such as peptide/antibody-based antiarrhythmic approaches, RNA interference, and immunotherapy (Lumley, 2002; Salanti et al., 2011; Boutjdir and Lazzerini, 2020), beta blockers (BBs) are regarded as first-line therapy for LQTS patients in the absence of obvious contraindications. The most common BBs include atenolol, propranolol, metoprolol, and nadolol, all of which are efficient at reducing the risk of cardiac events [CEs, e.g., syncope, aborted cardiac arrest (ACA), and sudden cardiac death (SCD)] in LQTS patients (Chatrath et al., 2004). However, a previous study has shown that different BBs exhibit various pharmacodynamic and pharmacokinetics, which may explain why nadolol is superior to its counterparts in the treating of LQT2 patients (Wilde and Ackerman, 2014). Out of the above-mentioned BBs, propranolol has furthermore been found to be the least efficient in preventing the recurrence of CEs (Abu-Zeitone et al., 2014). In addition, the effectiveness of BBs can be affected by different genotype of LQTS. The protective effect of BBs has been proposed to be highest in LQT1 patients, lower (albeit present) in LQT2 patients, and completely absent in LQT3 (Priori et al., 2004). Interestingly, the efficacy of BBs has also been shown to be associated with individual factors, such as gender, age, and corrected QT (QTc) intervals. Previous research has demonstrated that QTc, gender, and age are indispensable factors influencing the clinical course of LQTS patients (Vincent et al., 2009; Mazzanti et al., 2018). We therefore performed a network meta-analysis to assess how epidemiological factors influence the efficacy of BBs for CE risk reduction in LQT1-3 patients, in order to define more beneficial therapeutic strategies in these patients.

## Methods

### Search Strategy and Selection Criteria

This meta-analysis was carried out in accordance with the Preferred Reporting Items for Systematic Reviews and Meta-Analyses (PRISMA) Statement. The article protocol was registered on the PROSPERO International Prospective Register of Systematic Reviews (CRD42020179454). We searched for and collected relevant studies published between Jan 1, 1990, and April 30, 2020, from MEDLINE, PubMed, embase, and Cochrane Library (CENTRAL) databases. The keywords applied for computerized searching were atenolol, propranolol, metoprolol, nadolol, long QT syndromes, and beta-blockers. Additionally, manual searches were also carried out to identify potentially relevant literatures (Supplementary Table S1).

### Study Selection and Data Extraction

Studies included in this meta-analysis had to meet the following criteria: 1) The study selected participants with LQT1-3 genotypes. 2) Primary CEs included syncope, ACA, and SCD. 3) The studies needed to discuss the efficacy of different BBs on reducing the rate of CEs. 4) Articles needed to contain at least two of the following BBs: atenolol, propranolol, metoprolol, and nadolol. 5) The effectiveness of four BBs needed to be analyzed by comparing patients before and after BB therapy. 6) The data should be available for our analysis.

**FIGURE 1:**
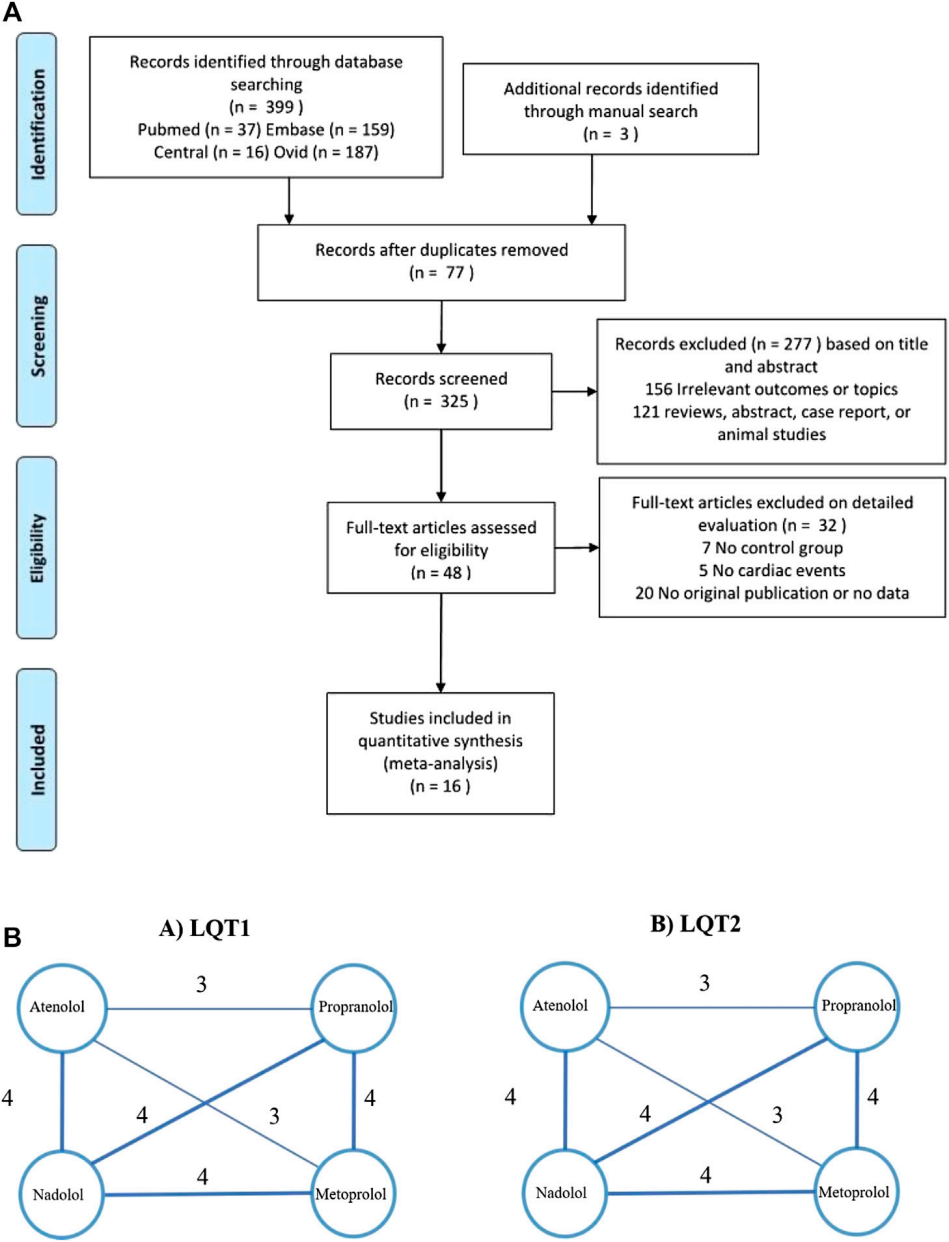
**(A)**, Flow diagram showing the identification of literatures according to the PRISMA format. **(B)**, Evidence of eligible comparisons for network meta-analysis. Numbers by the lines indicates the cumulative number of enrolled studies for each indirect comparison.

If papers met any of the following criteria, they were excluded: 1) Duplicated studies and reviews, conference papers, abstracts, and case reports. 2) Studies of subjects who received two or more BBs in combination therapy. 3) Studies that did not involve a comparison of efficacy among the four BBs. 4) Studies that provided insufficient and unqualified data (Figure 1A) (Moss et al., 2000; Conrath et al., 2002; Chatrath et al., 2004; Priori et al., 2004; Wang and Wu, 2004; Goldenberg et al., 2008; Shimizu et al., 2009; Vincent et al., 2009; Goldenberg et al., 2010; Chockalingam et al., 2012; Goldenberg et al., 2012; Abu-Zeitone et al., 2014; Koponen et al., 2015; Steinberg et al., 2016; Wilde et al., 2016; Mazzanti et al., 2018).

### Data Extraction and Quality Assessment

We extracted information from selected studies, including authors, study type, year of publication, race, number of participants with or without BBs, age, types of BBs, clinical CEs, the period of follow-up, gender, and the length of QTc interval (Table 1). Quality assessment was carried out by two independent reviewers using a standardized data collection form (Stang et al., 2018), as presented in Supplementary Figure S1. Discrepancies were addressed and resolved by a third reviewer. Sensitivity analysis was conducted to verify the robustness of the above results and to evaluate the deviational degree of each study; sensitivity analysis was performed using the command metaninf in STATA 12.0 (Stata Corp, College Station, TX). Rank probabilities were calculated via the surface under the cumulative ranking curve (SUCRA) (Fabritz et al., 2010). The SUCRA line shows the effectiveness of each treatment accounting for all possible rankings.

**TABLE 1:** Characteristics of included studies according to the effective of BBs on CEs.

Study (Ref. no)	Study design	No. of participant (female,%)	The percentage of treated with BBs, %	Prescribed BBs and dose	Follow-up duration, yrs	Mean QTc value	Definition of CEs	Mean ages, yrs	Included LQTS genotype(s)
Moss et al. (2000)	ITS	869 (62.3)	100	Atenolol: 1.36 ± 0.8 (mg/kg/day)	5	Probands: 520 ± 60	Syncope, ACA,SCD	15.7	LQT1, LQT2, LQT3
				Metoprolol: 1.8 ± 1.1 (mg/kg/day)					
				Nadolol: 1.4 ± 1.0 (mg/kg/day)		Affected family members: 500 ± 40			
				Propranolol: 2.9 ± 1.8 (mg/kg/day)					
Conrath et al. (2002)	ITS	87 (females: 55.2, girls: 13.8, males: 16.0, boys: 15.0)	100	NS	5.5 ± 5.7	Boys, LQT1: 504 ± 10	NS	NS	LQT1, LQT2
						LQT2: 488 ± 9			
						Girls, LQT1: 485 ± 9			
						LQT2: 461 ± 19			
						Males, LQT1: 472 ± 10			
						LQT2: 494 ± 12			
						Females, LQT1: 502 ± 19			
						LQT2: 493 ± 8			
Chatrath et al. (2004)	Cohort	28 (71.4)	100	Atenolol, metoprolol, Nadolol: 0.75–1.25 mg/kg/day	4	507 ± 67	Syncope, ACA	19.8 ± 11.9	LQT1, LQT2, LQT3
				Propranolol: 2.5–4 mg/kg/day					
Priori et al. (2004)	ITS	335 (62)	100	Nadolol: 1.2 ± 0.5 mg/kg/day	4.7 (0.6–36)	492 ± 47	ACA, SCD	26 ± 17	LQT1, LQT2, LQT3
				Propranolol: 2.2 ± 1.04 mg/kg/day					
Wang and Wu (2004)	ITS	26 (65.3)	84.6	Metoprolol: 67.5 ± 39.1 mg/day	3.1 ± 1	560 ± 60	Syncope	19 ± 10	LQT1, LQT2
				Propranolol: 60.0 ± 5.5 mg/day					
Goldenberg et al. (2008)	Cohort	3,015 children (girls: 63)	21.3	Atenolol, female: 75 ± 51 mg/day	Female: 11.6 ± 1.7	Female: 493 ± 49	Syncope, ACA, SCD	7.5 ± 5.4	LQT1, LQT2, LQT3
				Male: 85 ± 61 mg/day					
				Metoprolol, female: 119 ± 72 mg/day					
				Male: 89 ± 55 mg/day					
				Nadolol, female: 57 ± 49 mg/day	Male: 489 ± 48	Male: 489 ± 48			
				Male: 50 ± 35 mg/day					
				Propranolol, female: 51 ± 35 mg/day					
				Male: 51 ± 34 mg/day					
Vincent et al. (2009)	ITS	216 (64)	100	Nadolol, 2.2 ± 1.1 mg/kg/day	12.5 (7–26)	495 ± 46	Syncope, ACA, SCD	26 (18–42)	LQT1
				Propranolol, 1.7 ± 0.79 mg/kg/day					
Shimizu et al. (2009)	Cohort	858 (27.8)	23.4	NS	41	Rochester: 490 ± 60	Syncope, ACA, SCD	Rochester: 25 ± 20	LQT2
						Netherlands: 470 ± 50		Netherlands: 33 ± 21	
						Japan: 490 ± 50		Japan: 30 ± 18	
						Mayo: 470 ± 50		Mayo: 22 ± 16	
Goldenberg et al. (2010)	Cohort	971 (58.7)	57.2	Atenolol: 49 ± 32 mg/day	LQT1: 5.0 ± 6.8	QTc ≥ 500 (34.5%)	Syncope, ACA, SCD	31 ± 12	LQT1, LQT2
				Metoprolol: 67 ± 55 mg/day					
				Nadolol: 58 ± 45 mg/day	LQT2: 6.1 ± 7				
				Propranolol: 96 ± 71 mg/day					
Goldenberg et al. (2012)	Cohort	721 (45.3)	43.8	NS	NS	CE triggered by four factor or no event	Syncope, ACA, SCD	30 ± 12	LQT1
						Exercise: 504 ± 52			
						Acute arousal: 491 ± 49			
						Sleep/rest nonarousal: 486 ± 58			
						Other triggers: 480 ± 48			
						No event: 471 ± 43			
Chockalingam et al. (2012)	Cohort	382 (56)	100	Metoprolol: 1.4 (0.9–2.5)	Propranolol: 2 (1–6)	472 ± 46	Syncope, ACA, SCD	14 (8–32)	LQT1, LQT2
				Nadolol: 0.8	Metoprolol: 4 (2–8)				
				Propranolol: 2.5 (1.3–3)	Nadolol: 3 (2–5)				
Abu-zaitone et al. (2014)	Cohort	1,530 (60.1)	45.1	Atenolol, Age>18 years: 0.7 ± 0.3	5	Atenolol: 492 ± 49	Syncope, ACA, SCD	14.9	LQT1, LQT2
				Age<18 years: 1.0 ± 0.7					
				Metoprolol, Age>18 years: 1.2 ± 0.9		Metoprolol: 496 ± 52			
				Age<18 years: 1.4 ± 1.0					
				Nadolol, Age>18 years: 1.0 ± 0.8		Nadolol: 490 ± 51			
				Age<18 years: 1.0 ± 0.8					
				Propranolol, Age>18 years: 2.1 ± 2.3		Propranolol: 500 ± 58			
				Age<18 years: 2.3 ± 1.5					
Koponen et al. (2015)	Cohort	316 (53.1)	77.2	Metoprolol: 1.3 ± 0.4 (mg/kg/day)	5.8	LQT1 mutation (G589D): 454 ± 35	Syncope, ACA, SCD	12.0 ± 5.5	LQT1, LQT2
						Mutation (c.1129–2A > G): 465 ± 37			
				Bisoprolol: 0.1 ± 0.1 (mg/kg/day)		Non-mutation: 475 ± 43 LQT2			
				Atenolol: 1.2 ± 1.5 (mg/kg/day)		Mutation (L552S): 448 ± 32			
				Propranolol: 2.4 ± 0.8 (mg/kg/day)		Mutation (R176W): 436 ± 31			
						Non-mutation: 480 ± 39			
Steinberg et al. (2016)	Cohort	114 (58.7)	100	Atenolol: 53 ± 30 mg/day	Atenolol: 6 (3–10)	Atenolol: 466 ± 23	Syncope, ACA, VT	Atenolol: 37 ± 19	LQT1, LQT2
				Nadolol: 74 ± 47 mg/day	Nadolol: 3 (1–5)	Nadolol: 469 ± 32		Nadolol: 27 ± 13	
Wilde et al. (2016)	Cohort	391 (55)	29	ND	7.25	476 ± 57	Syncope, ACA, SCD	28 ± 20	LQT3
Mazzanti et al. (2018)	Cohort	1710 (52)	ND	Nadolol: >0.5 mg/kg/day	9 ± 7	471 ± 45	Syncope,ACA, SCD	ND	LQT1, LQT2, LQT3
				Propranolol: >1.5 mg/kg/day					
				Metoprolol: >1.0 mg/kd/day					

BBs: beta-blockers; LQTS: long-QT syndromes; ITS: interrupted time series; ACA: aborted cardiac death; SCD:sudden cardiac death; VT: ventricular tachycardia.

### Statistical Analysis

Hazard ratios (HRs) with 95% confidence intervals (CIs) of CEs were extracted or analyzed. We conducted our meta-analysis by applying random-effects models. HRs were assessed using the Inverse-Variance method, as well as the calculation of subgroups. Heterogeneity among these comparisons was evaluated using the *I* ^2^ test, with an *I* ^2^ > 75% considered as a series of comparisons with unacceptable heterogeneity. Statistical calculations in traditional meta-analyses were performed using RevMan 5.3 (Cochrane Collaboration, Oxford, United Kingdom) and STATA 12.0 software. Moreover, a network meta-analysis was evaluated using a random-effects model within a Bayesian framework (Lumley, 2002). HRs and corresponding 95% credible intervals (CrIs) were analyzed using gemtc package (https://drugis.org/software/r-packages/gemtc) in R (x64 3.6.0) for all statistical analyses. Publication bias was evaluated using the command metabias with the Egger’s linear regression test in STATA 12.0. When the number of studies was less than 10, the Egger’s linear regression test was used to measure the publication bias by performing a quantitative test on the funnel chart.

## Results

### Correlation Between Gender, Age, Corrected QT, and CEs Risk in Long QT Syndrome Patients

**FIGURE 2:**
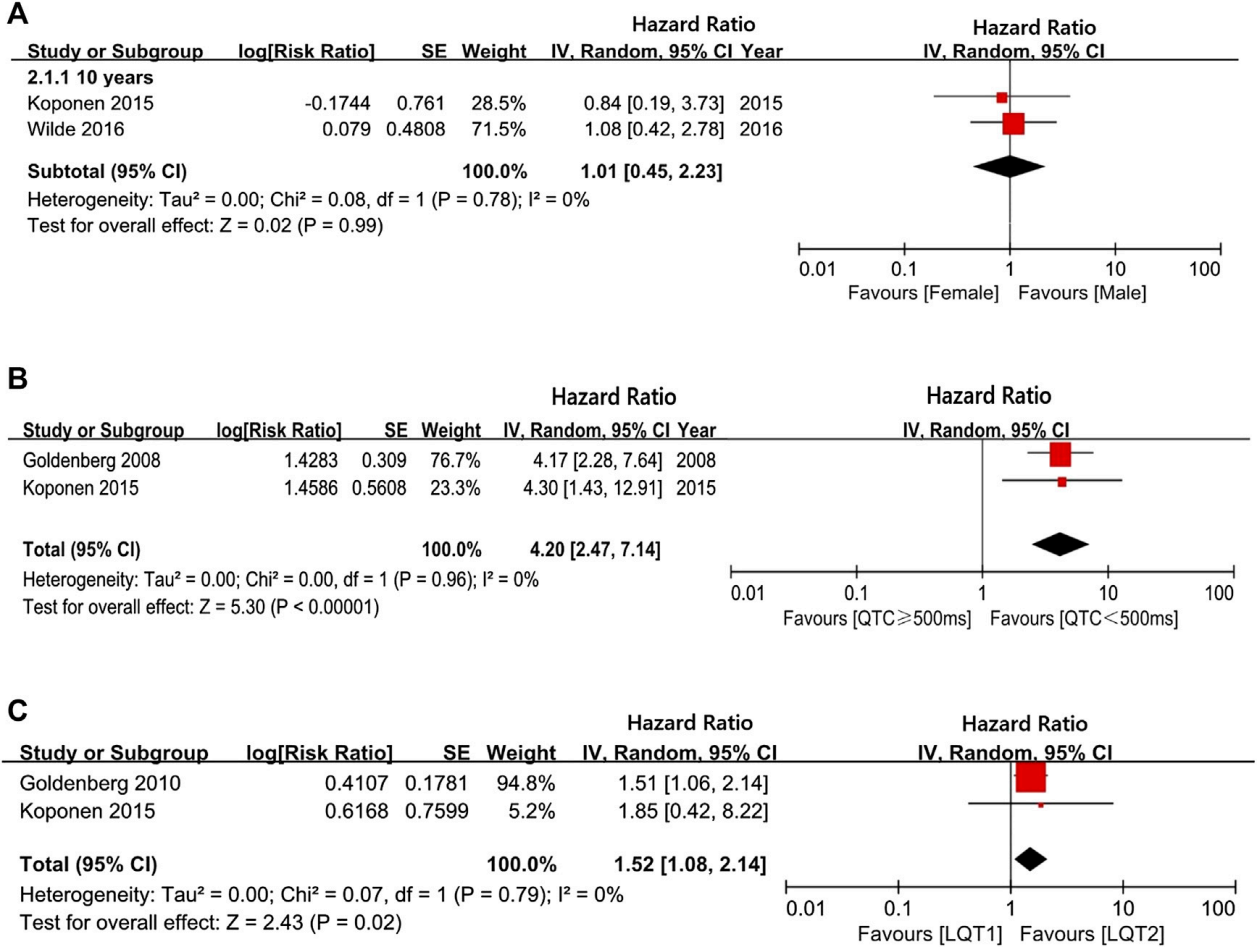
The risk of CEs in a long follow-up period. **(A)**, the hazard ratio (HR) values in comparing females/males at 10-years-old; **(B)**, the HR values in comparing QTc ≥ 500 ms/QTc < 500 ms at 10-years-old; **(C)**, the HR values in comparing LQT1/LQT2 at 10-years-old.

Our analysis showed that before puberty, male and female LQTS patients had a similar likelihood of experiencing CEs (girls vs. boys in group of 10-years-old: HR 1.01, 95% CI 0.45–2.23; *I* ^2^ = 0%, *p* for heterogeneity 0.78; Figure 2A). However, the risk of CEs between female and male LQTS patients after the onset of puberty was not compared due to a lack of data. Individuals with a QTc ≥ 500 ms had a higher risk of CEs than individuals with QTc < 500 ms during childhood (HR 4.20, 95% CI 2.47–7.14, *I* ^2^ = 0%, *p* = 0.96; Figure 2B). On the other hand, CE risk was higher in LQT1 patients than in LQT2 patients at the age of 10 (HR 1.52, 95% CI 1.08–2.14; *I* ^2^ = 0%, *p* = 0.79; Figure 2C). Due to the lack of sufficient data throughout the adolescent period, there was no assessment of different QTc intervals and genotypes on CE risk in this age group.

#### The Efficacy of Beta Blockers in Long QT Syndrome Patients Based on the Different Gender, Age, and Corrected QT Intervals

**FIGURE 3:**
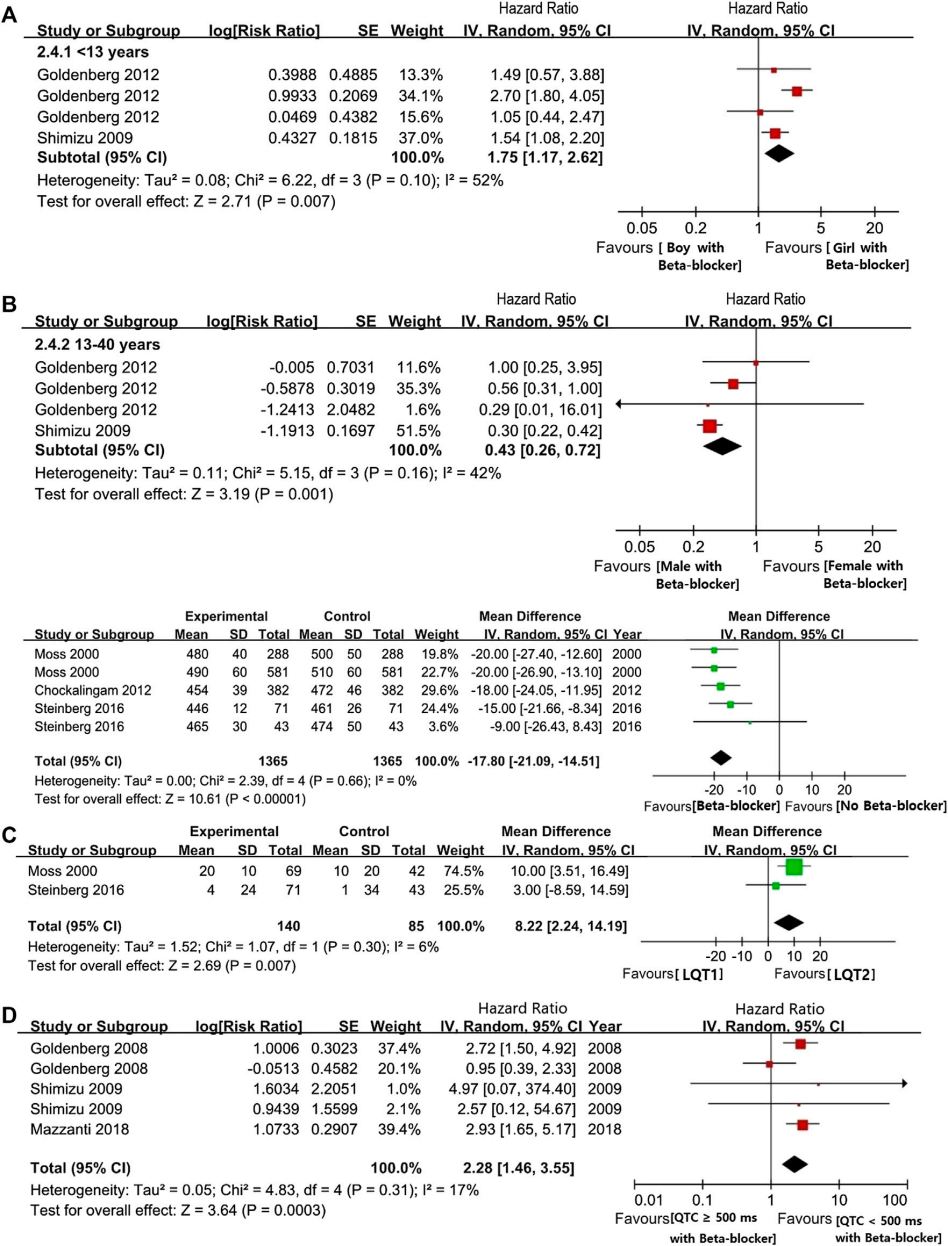
**(A)**, The HR value of CEs in LQTs patients with female and male. **(B)**, the HR value of CEs via comparsing female and male patients during pre-adolescent or post-adolescent periods (males vs. females). **(C)**, The efficacy of BBs on shorting the length of QTc intervals. **(D)**, The efficacy of BB on shorting the length of QTc intervals in comparison with LQT1 and LQT2 (LQT1 vs. LQT2). **(E)**, The HR value of the possibility of CEs in the comparsion between LQTs patients with QTc ≥ 500 ms or QTc < 500 ms (QTc ≥ 500 ms vs. QTc < 500 ms).

Overall, BBs treatments showed a significant risk reduction for CEs in LQTS patients. Throughout preadolescence, boys were more likely to be affected by CEs than girls when both were treated with BBs (boys vs. girls: HR 1.75, 95% CI 1.17–2.62; *I* ^2^ = 52%, *p* = 0.1; Figure 3A). Interestingly, after puberty, females had a higher likelihood of developing CEs than male patients, despite BB therapy (males vs. females, ages 13–40: HR 0.43, 95% CI 0.26–0.72 *I* ^2^ = 42%, *p* = 0.16; Figure 3A). Thus, we observed that age and gender could synergistically influence the efficacy of BB therapy on the risk reduction of CEs.

Our findings showed that BBs therapy efficiently decreased QTc intervals lengths by an average of 17.8 ms (95% CI 14.51–21.09 ms; Figure 3B). Under BBs treatments, patients with the LQT1 genotype had a significantly higher reduction in QTc-length than LQT2 patients (95% CI 2.24–14.19 ms, *I* ^2^ = 6%, *p* = 0.30; Figure 3C). Furthermore, similarly to the above findings, even after BBs therapy, LQTS patients with QTc ≥ 500 ms had a greater risk of experiencing CEs than subjects with QTc < 500 ms (HR 2.28, 95% CI 1.46–3.55; *I* ^2^ = 17%, *p* = 0.31, Figure 3D).

#### Comparison of the Efficiency of Different Beta Blockers in the Three Main LQT Genotypes

**FIGURE 4:**
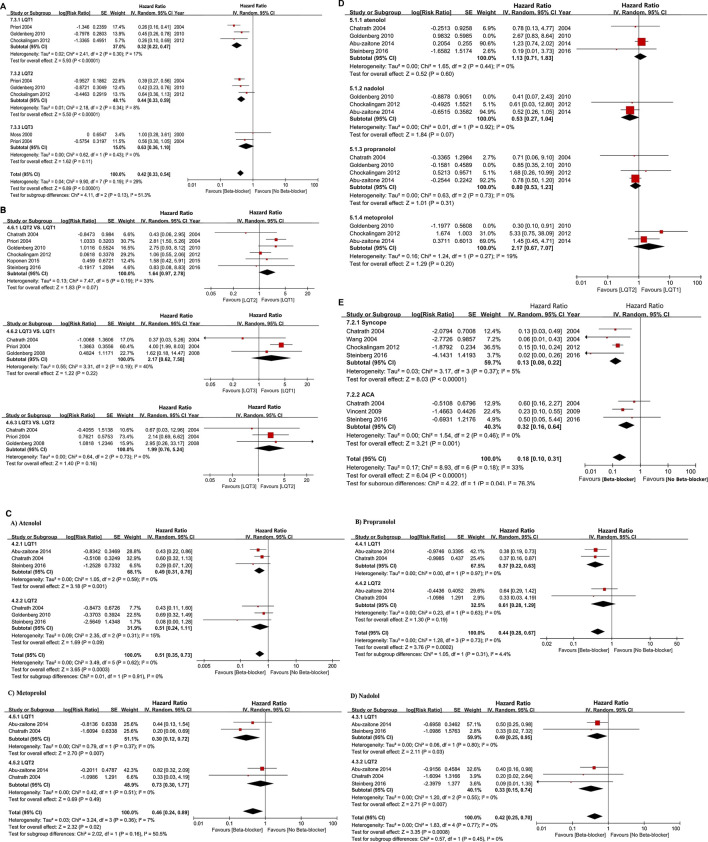
**(A)**, The HR value of CEs in comparing the two different LQT genotypes, including LQT2 vs. LQT1, LQT3 vs. LQT1, and LQT3 vs. LQT2. **(B)**, the effectiveness of BBs in reducing the occur of CEs via comparsing LQT2 and LQT1 (LQT2 vs. LQT1). **(C)**, The efficacy of different BBs on the risk reduction of CEs in LQT1 and LQT2. **(D)**, The efficacy of BBs therapy on managing of CEs in different LQTs genotypes, including LQT1, LQT2, LQT3. **(E)**, The efficacy of BBs therapy in reducing the incidence of syncope or ACA respectively.

We observed a significant reduction of CEs in LQT1 and LQT2 following BBs management, but not in LQT3 (LQT1: HR 0.32, 95% CI 0.24–0.47; *I* ^2^ = 17%, *p* = 0.3; LQT2: HR 0.44, 95% CI 0.33–0.59; *I* ^2^ = 8%, *p* = 0.34; LQT3: HR 0.63, 95% CI 0.36–1.10; *I* ^2^ = 0%, *p* = 0.43; Figure 4A). Upon intervention with BBs, LQT2 patients tended to have a greater propensity of suffering CEs compared to LQT1 patients, although this was not statistically significant (LQT2 vs. LQT1: HR 1.64, 95% CI 0.97–2.78; *I* ^2^ = 33%, *p* = 0.19; Figure 4B). Interestingly, when the effect of BBs in LQT3 was respectively compared with LQT2 or LQT1 alone, it revealed a similar efficacy for controlling CE risk (LQT3 vs. LQT1: HR 2.17, 95% CI 0.62–7.58; *I* ^2^ = 40%, *p* = 0.19 and LQT3 vs. LQT2: HR 1.99, 95% CI 0.76–5.24; *I* ^2^ = 0%, *p* = 0.73; Figure 4B).

Atenolol appeared to reduce the risk of CEs in LQT1, but not in LQT2 patients (HR 0.49, 95% CI 0.31–0.76; *I* ^2^ = 0%, *p* = 0.59 in LQT1 and HR 0.51, 95% CI 0.24–1.11; *I* ^2^ = 15%, *p* = 0.31 in LQT2; Figure 4C). However, comparing these groups directly, we found in significant differences in the efficacy of atenolol between LQT1 and LQT2 patients (LQT2 vs. LQT1, HR 1.13, 95% CI 0.71–1.83; *I* ^2^ = 0%, *p* = 0.44; Figure 4D). Propranolol also attenuated the rate of CEs in patients with LQTS (HR 0.44, 95% CI 0.28–0.67; *I* ^2^ = 0%, *p* = 0.73; Figure 4C). Although the efficiency of propranolol appeared to be somewhat higher in LQT2 than in LQT1 patients, these results also did not reach significance (LQT2 vs. LQT1, HR 0.80, 95% CI 0.53–1.23; *I* ^2^ = 0%, *p* = 0.73; Figure 4D). Metoprolol exhibited an obvious risk-reducing effect in LQT1 (HR 0.30, 95% CI 0.12–0.72; *I* ^2^ = 0%, *p* = 0.37; Figure 4C), but not in LQT2 (HR 0.73, 95% CI 0.30–1.77; *I* ^2^ = 0%, *p* = 0.51; Figure 4C), although a direct comparison of the efficacy of this BB in LQT1 and LQT2 patients was not statistically significant (LQT2 vs. LQT1, HR 2.17, 95% CI 0.67–7.07; *I* ^2^ = 19%, *p* = 0.27; Figure 4D). Nadolol provided a strong risk reduction for CEs in LQTS patients (HR 0.42, 95% CI 0.25–0.70; *I* ^2^ = 0%, *p* = 0.77; Figure 4C). This effect was more pronounced in LQT2 than in LQT1 patients, and a direct comparison between the groups revealed that this was statistically significant (LQT2 vs. LQT1, HR 0.53, 95% CI 0.27–1.04; *I* ^2^ = 0%, *p* = 0.92; Figure 4D). We observed a different efficacy of these BBs for risk reduction of different types of CEs. BBs seemed to be more effective at preventing syncope than decreasing the rate of ACA (HR 0.13, 95% CI 0.08–0.22; *I* ^2^ = 5%, *p* = 0.37 in syncope and HR 0.32, 95% CI 0.16–0.64; *I* ^2^ = 0%, *p* = 0.46 in ACA; Figure 4E).

#### Joint Comparison of the Effectiveness of Different Beta Blockers

**FIGURE 5:**
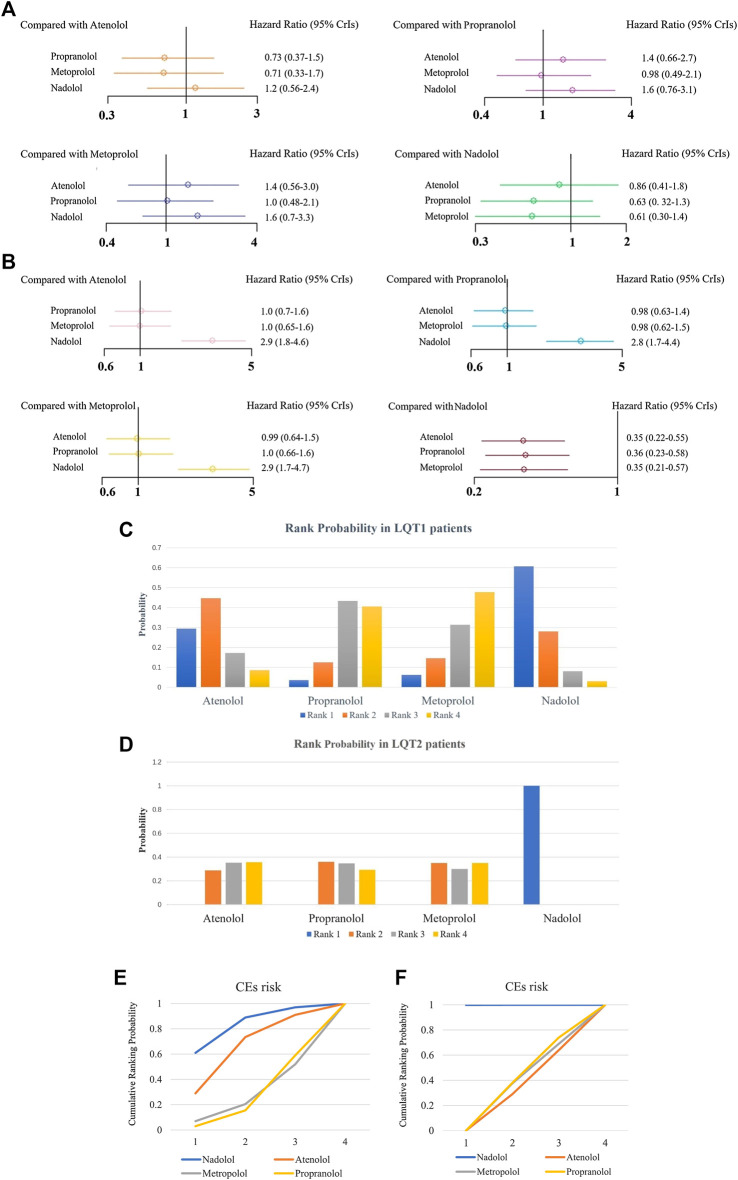
**(A, B)**, The effect of different BBs on risk reduction of CEs in patients with LQT1 **(A)** or LQT2 **(B)**. **(C, D)**, The rank probability of BBs therapy for reducing the occurrence of CEs in LQT1 **(C)** and LQT2 **(D)** patients. E-F, SUCRAs between 0 and 1 represent the probability of being ranked highest. For the CEs risk, higher score corresponds to higher proportion achieving at least 5% CEs risk reduction with a most effective therapy in LQT1 **(E)** and LQT2 **(F)** patients.

For LQT1 patients, the CrIs value was too wide to show significance for the difference observed for atenolol over propranolol and metoprolol (atenolol vs. propranolol, HR 0.73, 95% CrIs 0.37–1.5, and atenolol vs. Metoprolol, HR = 0.71, 95% CrIs 0.33–1.7; Figure 5A, Table 2). Conversely, atenolol efficacy for risk reduction seemed somewhat lower than for nadolol (atenolol vs. nadolol, HR = 1.2, 95% CrIs 0.56–2.4; Figure 5A; Table 2). Propranolol and metoprolol had similar efficacy for risk reduction (propranolol vs. Metoprolol, HR 0.98, 95% CrIs 0.49–2.1; Figure 5A; Table 2). All four BBs interventions had an almost equal effect in LQT1 patients via pairwise comparisons (nadolol vs. propranolol: HR 0.63, 95% CrIs 0.32–1.3; and nadolol vs. Metoprolol: HR 0.61, 95% CrIs 0.3–1.4; Figure 5A; Table 2). In addition, we found that LQT2 patients treated with nadolol showed the greatest decrease in the risk of CEs compared to the other three BBs (nadolol vs. atenolol: HR 0.35, 95% CrIs 0.22–0.55; nadolol vs. propranolol: HR 0.36, 95% CrIs 0.23–0.58 and nadolol vs. Metoprolol: HR 0.35, 95% CrIs 0.21–0.57; Figure 5A; Table 2). With the exception of nadolol, pairwise comparisons among the other three BBs exhibited no superiority to any others in managing LQT2 patients (atenolol vs. propranolol: HR 1.0, 95% CrIs 0.7–1.6; atenolol vs. Metoprolol: HR 1.0, 95% CrIs 0.65–1.6 and propranolol vs. Metoprolol: HR 0.98, 95% CrIs 0.62–1.5; Figure 5A; Table 2). Due to a lack of sufficient data, we were unable to assess the effectiveness of different BBs for LQT3 patients.

**TABLE 2:** The efficacy of different BB therapies on reducing the occurrence of CEs (syncope, SCD, ACA) in patients with LQT1 and LQT2 by the network meta-analysis using HR and 95% CrIs.

LQT1				
Cardiac events	Atenolol	1.4 (0.66–2.7)	1.4 (0.56–3.0)	0.86 (0.41–1.8)
	0.73 (0.37–1.5)	Propranolol	1.0 (0.48–2.1)	0.63 (0.32–1.3)
	0.71 (0.33–1.7)	0.98 (0.49–2.1)	Metoprolol	0.61 (0.3–1.4)
	1.2 (0.56–2.4)	1.6 (0.76–3.1)	1.6 (0.7–3.3)	nadolol
**LQT2**				
Cardiac events	Atenolol	0.98 (0.63–1.4)	0.99 (0.64–1.5)	0.35 (0.22–0.55)
	1.0 (0.7–1.6)	Propranolol	1.0 (0.66–1.6)	0.36 (0.23–0.58)
	1.0 (0.65–1.6)	0.98 (0.62–1.5)	Metoprolol	0.35 (0.21–0.57)
	2.9 (1.8–4.6)	2.8 (1.7–4.4)	2.9 (1.7–4.7)	nadolol

#### Relative Ranking of Four Beta Blockers

Next, we used SUCRA to analyze rank probability of different BBs on the risk reduction for CEs based on LQTS genotype, as shown in Table 3 and Figures 5C–F. For LQT1 patients, nadolol ranked first with a higher efficacy for reducing CEs risk (Figures 5C,E). Interestingly, we noticed that atenolol was the second-most effective treatment of the four BBs for patients with LQT1. Propranolol was ranked third, and metoprolol was ranked last. In LQT2 patients, nadolol was further verified to be a first-line therapy with minimal risk of CEs. The other three BBs treatments displayed no benefit for managing the CEs risk (Figures 5D,F).

**TABLE 3:** Relative ranking of different BBs assessed by using SUCRA values.

	Rank 1	Rank 2	Rank 3	Rank 4
LQT1				
Atenolol	0.294350	0.447550	0.17180	0.0863
Propranolol	0.035675	0.124925	0.43360	0.4058
Metoprolol	0.062550	0.146400	0.31355	0.4775
Nadolol	0.607425	0.281125	0.08105	0.0304
LQT2				
Atenolol	0.000250	0.289075	0.352525	0.358375
Propranolol	0.000150	0.36075	0.347125	0.291975
Metoprolol	0.000100	0.349900	0.300350	0.349650
Nadolol	0.999725	0.000275	0.000000	0.000000

## Discussion

The aim of this study was to assess the relationship between epidemiological variables (gender, age, and QTc intervals) and CE risk in LQTS patients using a meta-analysis. We furthermore systematically evaluated the efficacy of BBs therapy in LQTS with different ages, genders, and QTc intervals, as well as genetic subtypes (Bohnen et al., 2017). It is well-established that the rate of CEs in LQTS patients is closely correlated with age and sex (Wang and Wu, 2004). Previous reports have indicated that the risk of fatal events in LQTS is higher in boys than girls during childhood (Goldenberg et al., 2008). After adolescence, gender-related risk is reversed, and a greater risk of CEs is observed in the group of female patients (Goldenberg et al., 2012). This might be due to longer QTc intervals in LQTS female patients than in male patients in adulthood (Conrath et al., 2002). However, we showed that boys and girls with LQTS have a similar risk of CEs during pre-adolescence. After the onset of puberty, there was no result concerning the risk of CEs among females’ and males’ patients due to a lack of data (Figure 2A). Strikingly, although there was a decreased risk for CEs in both males and females after BBs treatment regardless of age, girls were less likely to experience CEs than boys in pre-puberty (<13-year-old). After the onset of adolescence, BBs were more efficient at reducing CEs in males than in females (13–40-year-old) (Figure 3A).

Recent studies have suggested that both genotype and QTc duration are independent risk factors influencing the risk of CEs in patients with LQTS (Mazzanti et al., 2018; Priori et al., 2004; Ebbehøj et al., 2004). Furthermore, a 3.3-times lower risk for CEs were detected in LQTS patients with shorted QTc duration compared to patients with prolonged QTc duration (Koponen et al., 2015). Consistent with this view, our result indicates that LQTS patients with QTc intervals ≥500 ms had a higher risk of experiencing CEs compared to patients with QTc < 500 ms during childhood (at 10-years-old) (Figure 2B). Unfortunately, due to insufficient data in the present studies, we were unable to assess the effect of QTc duration on the risk of CEs in adulthood. The efficacy of BBs in patients with QTc ≥ 500 ms was lower than in patients with QTc < 500 ms (Figure 3D). Our study found that BBs reduce the QTc interval in LQTS patients by an average of 17.8 ms (Figure 3B). We also observed that this effect of decreasing QTc was more pronounced in LQT1 patients than in LQT2 patients (Figure 2C). A previous study showed a 3-fold increased risk for CEs in LQT2 patients compared to LQT1 during adulthood, regardless of BBs therapy (Goldenberg et al., 2010). Interestingly, we summarized that, in the pre-adolescent period, LQT1 patients were reported to have more CEs than LQT2 patients. The rate of CEs among LQT1 and LQT2 patients in post-adolescence was not presented due to a lack of sufficient evidence (Figure 2C). Thus, appropriate stratification in terms of the above factors should be performed, in order to guide clinical decision-making for BBs therapy and improve the prognosis of LQTS with the minimum adverse-events (Migdalovich et al., 2011; Westphal et al., 2020).

BBs are considered the most effective therapy for alleviating CEs in LQTS patients (Moss et al., 2000). Notably, although BB treatments may be applied to LQT1 patients with a minimum risk of CEs during a long follow-up period, this does not mean that it is inherently safe for treating patients with other LQT subtypes with the same BB (Postema et al., 2013). Therefore, it is important to investigate which BBs perform best for controlling CEs in different LQTS genotypes. In our study, we found that after BBs therapy, patients with LQT1 had a relatively lower rate of CEs than patients with LQT2 (Figure 4B). BBs therapy had no apparent function in decreasing CEs risk for LQT3 patients, but the risk of CEs in LQT3 patients was generally higher than in other LQTS genotypes (Priori et al., 2004). This phenomenon could be explained by the incorrect notion that BBs therapy has no effect on LQT3 patients (Schwartz et al., 2012) (Figure 4A). Strikingly, the blocking effect of BBs is observed among LQT3 female patients but absent in male patients (Wilde et al., 2016). We confirmed a protective effect of BBs therapy for LQT2 and LQT3 patients was somewhat lower than for LQT1 patients, which was consistent with prevailing findings (Shimizu et al., 2009), although there was no statistically significant difference on the risk of CEs via the pairwise comparisons between the LQT1-3 genotypes (Figure 4B). Among the four BBs, we investigated that nadolol exhibited a pronounced risk reduction in both LQT1 and LQT2 (HR 0.49 and HR 0.33, respectively; Figure 4C). However, atenolol, propranolol, and metoprolol only prevented LQT1 patients from CEs (HR 0.49, 0.37 and 0.30, respectively), but did not prevent them in LQT2 patients (HR 0.51, 0.61 and 0.73, respectively). Nadolol, a hydrophilic long-acting nonselective drug with the longest elimination half-life of these four BBs, could maintain high pharmacodynamic levels, which might be the main reason why it is regarded as the most effective BB therapy for LQTS patients (Abu-Zeitone et al., 2014). In addition, it could be because of the membrane-stabilizing effect of nadolol, which was attributed to its effect of shorting QTc intervals when compared to atenolol, propranolol and metoprolol in LQT2 patients (Chockalingam et al., 2012). On the other hand, nadolol exhibited a significant effect of reducing the CEs in LQT2. Compared to the effect of BBs between LQT1 and LQT2 patients, there was no superiority in controlling the recurrence of CEs among other three BBs, including atenolol, propranolol, and metoprolol (Figure 4D). Lastly, we also observed that BBs seemed to be somewhat more effective in preventing syncope than ACA in LQTS patients (Figure 4E).

**FIGURE 6:**
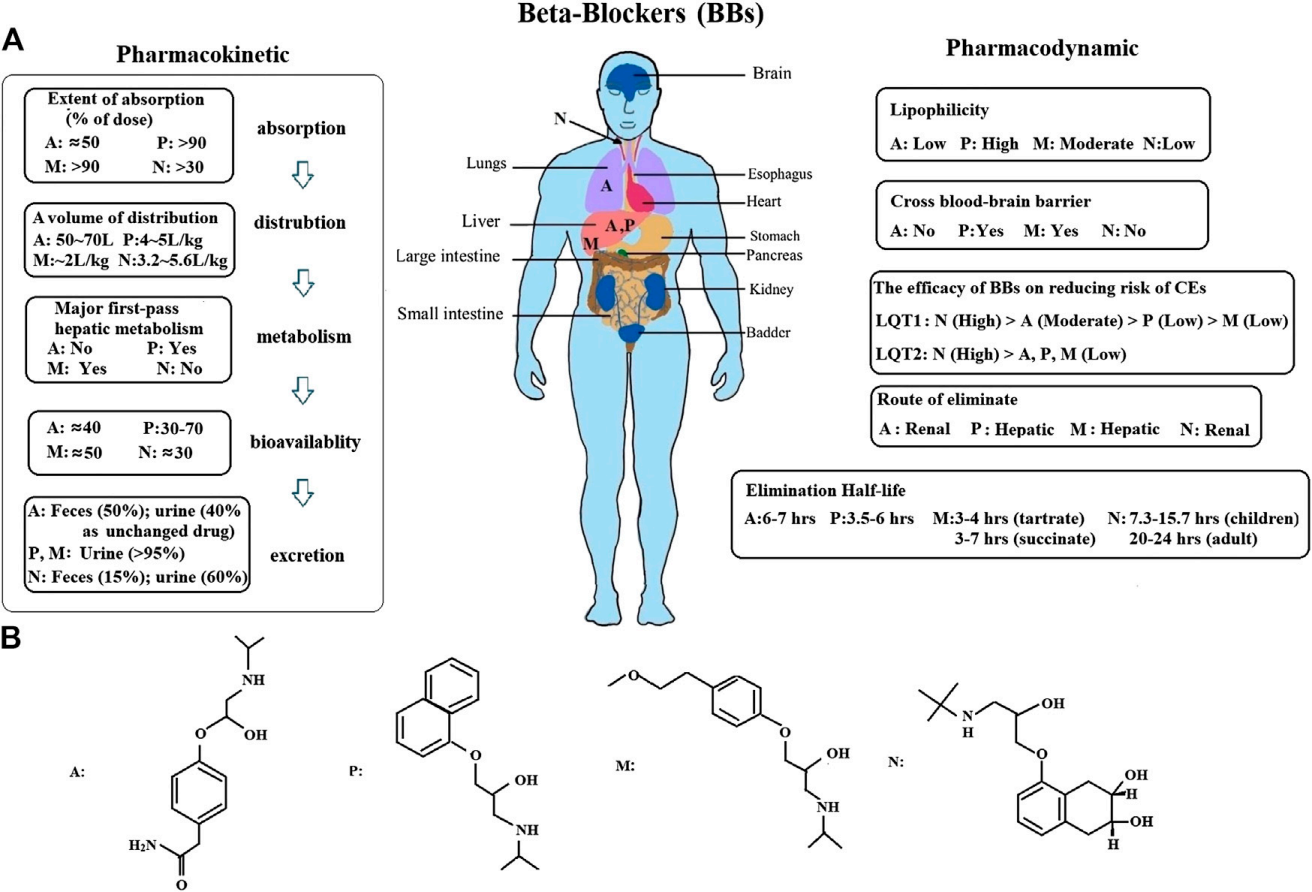
**(A)**, An overview of the pharmacokinetic and pharmacodynamic of BBs was presented. Atenolol is mainly concentrated in the lungs and liver. Propranolol and metoprolol are almost totally metabolized in the liver. Nadolol is absorpted by the gastrointestinal tract, and primarily appears in blood serum. However, it is not metabolized by the liver in humans, only excreted in an unchanged form by the kidney (75%) (A: atenolol, P: propranolol, M: metoprolol, N: nadolol). **(B)**, The structural formula of BBs.

Furthermore, we conducted a network meta-analysis to assess the efficacy of different BBs in LQT1 and LQT2 via pairwise comparisons, and then joint ranked those results using cluster analysis. Steinberg et al. suggested that all four BBs had a similar effect in preventing the rate of CEs in LQT1, but nadolol was regarded as the most effective drug with the minimum risk of CEs in LQT2 (Steinberg et al., 2016). Our study also showed that nadolol had the best efficacy for reducing the risk of CEs in LQT2. Interestingly, it also ranked first for LQT1 with the lowest possibility of CEs. A previous article suggested that metoprolol had a greater risk of CEs in symptomatic patients than a combined therapy of propranolol and nadolol (Chockalingam et al., 2012). We retrieved various studies and our results have demonstrated that metoprolol is the least effective of the four studied BBs in decreasing the risk of CEs in LQTS (Figures 5A,B). The therapeutic effect of atenolol seemed somewhat superior compared to propranolol for LQT1 patients, but neither of them were beneficial in LQT2 (Figures 5A,B). Interestingly, there has been a controversial view on whether propranolol is inferior to its counterparts in high-risk LQTS patients (Steinberg et al., 2016; Kwok et al., 2017). It is well-known that the discrepancy of BBs efficacy in monitoring LQTS could be attributed to inadequate dosage and/or patients’ noncompliance in earlier research (Moss et al., 2000). In summary, our results propose that the ranked effectiveness of BBs in reducing CEs risk in LQT1 patients is the following: nadolol, atenolol, propranolol, and metoprolol. For LQT2 patients, nadolol showed a protective effect, while other BBs did not significantly prevent the occurrence of CEs, including atenolol, propranolol, and metoprolol (Figures 5C,D). These findings are consistent with previous reports (Ahn et al., 2017; Wallace et al., 2019). As described earlier, atenolol had fewer neuropsychiatric side effects, which was attributed to its lower lipid solubility and permeability of the blood-brain barrier (Chatrath et al., 2004). The above results indicate that if patients do not tolerate nadolol, atenolol could represent an alternative therapy for controlling CEs in LQT1 patients. However, for LQT2, propranolol might be a relatively better choice. The different efficacies of BBs was primarily due to the pharmacological and pharmacokinetic characteristics of each blocker (Ågesen et al., 2019) (Figure 6). Generally, long-term safety and effectiveness have to be considered for BB treatments in the clinical management of LQTS patients.

## Conclusion

In the present study, we investigated the relationship between the risk of CEs in LQTS patients and their age, gender, and QTc length. We also clarified different efficacies of BBs for CE risk reduction based on patients with LQTS genotypes. Our analysis did not only induce a pairwise comparison to reveal the efficacy of four BBs in LQTS patients, but also provided the ranked efficacies of BBs treatment for preventing the recurrence of CEs in LQT1 and LQT2 patients. Our results demonstrated that nadolol was the most effective therapy for LQT2 patients. However, in LQT1 patients, the effect of nadolol was also relatively superior to treatment with the three other BBs. In the future, we will investigate which BB is to be preferred for the management of LQTS patients with increased risk factors, such as QTc > 500 ms, male gender in pre-puberty, female gender in adulthood, and LQT2/LQT3 genotypes.

## Limitations

We only included sixteen studies, which was due to a lack of sufficient evidence reflecting the efficiency of BB treatments in LQTS patients based on randomized controlled trials. The results of sensitivity analysis indicated that studies had comparable bias and heterogeneities (Goldenberg et al., 2008). In addition, we will continue to retrieve new studies in order to further investigate the effectiveness of different BBs treatments in relation to other characteristics of LQTS patients, such as ethnicity. Finally, the BBs dosage and the follow-up years of patients plays a pivotal role in their efficacy (Ahrens-Nicklas et al., 2009). How those factors affect their efficacy in LQTS patients warrants further study.

## Author Contributions

LH and JL designed and performed this study. LH and FL independently used the inclusion disciplines to select identified and qualified literature, and extracted the data from it. QL, QT, and ZZ contributed to the process of double identifying and retrieving the studies from collected literatures. LH conducted traditional meta-analysis and network analysis. LH and JL. wrote and reviewed the draft. LH and ZX critically revised the manuscript.

## Funding

This work was supported in part by grants from the National Natural Science Foundation of China (No: 81760065), the Natural Science Foundation of Jiangxi Province (No: 20152ACB20025), and the Science and Technology Support of Jiangxi Province (No: 20151BB-G70166).

## Conflict of Interest

The authors declare that the research was conducted in the absence of any commercial or financial relationships that could be construed as a potential conflict of interest.
