# Supplementary figures and images for: The Efficacy of Beta-Blockers in Patients With Long QT Syndrome 1–3 According to Individuals’ Gender, Age, and QTc Intervals: A Network Meta-analysis
Source: Front Pharmacol. 2020 Dec 14;11:579525. doi: 10.3389/fphar.2020.579525 (PMC7768040; doi:10.3389/fphar.2020.579525)

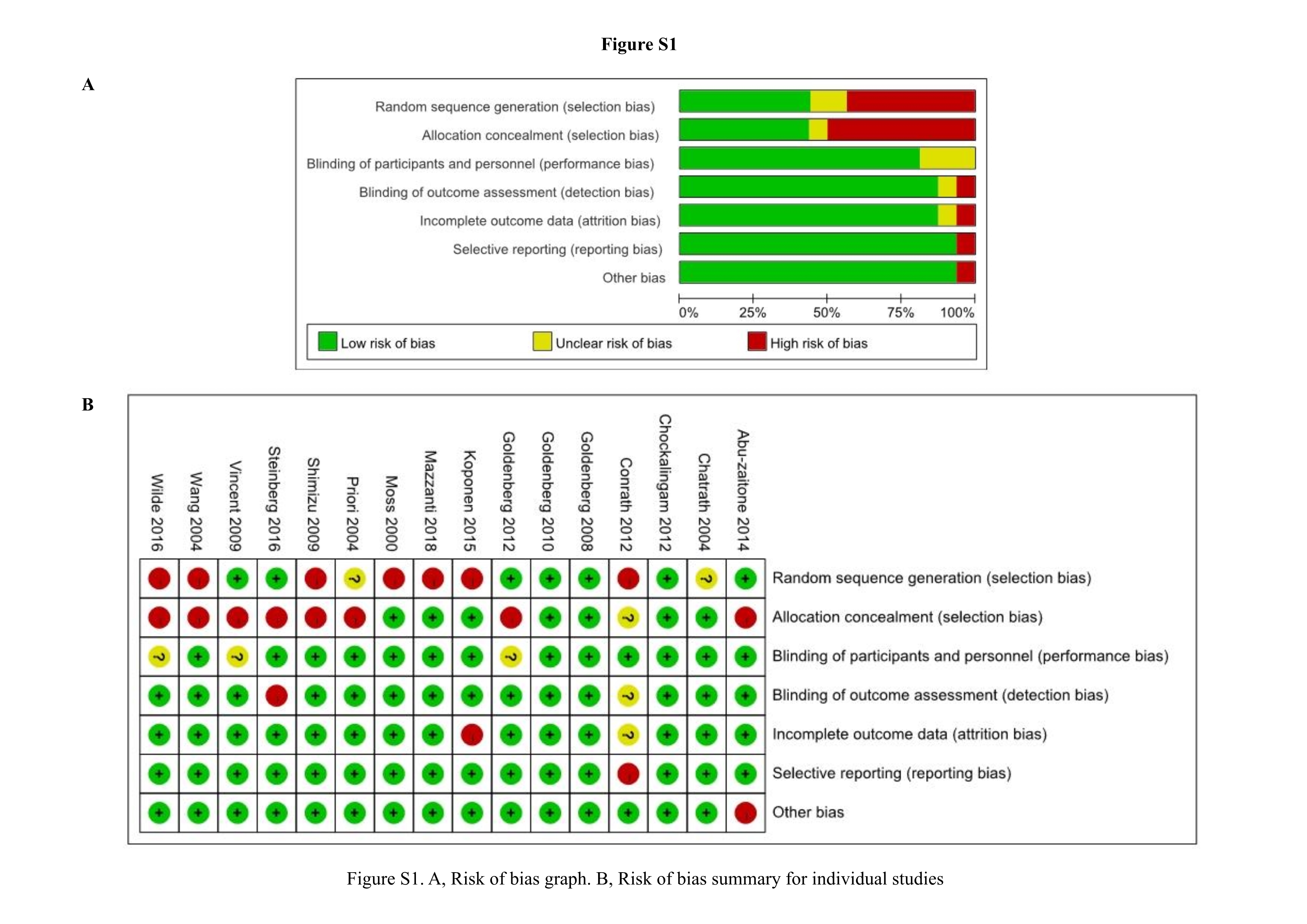

Supplement: Supplementary file 1 [file image1.jpeg]
