# Supplementary material for: The Efficacy of Beta-Blockers in Patients With Long QT Syndrome 1–3 According to Individuals’ Gender, Age, and QTc Intervals: A Network Meta-analysis
Source: Front Pharmacol. 2020 Dec 14;11:579525. doi: 10.3389/fphar.2020.579525 (PMC7768040; doi:10.3389/fphar.2020.579525)
Supplement: Supplementary file 2 [file table1.pdf]

Table S1. Database search strategy

#1 Beta-blockers

- Metoprolol
  'Toprol' OR 'Betaloc' OR 'Betaloc-Astra' OR 'Betaloc Astra' OR 'Betalok' OR 'CGP-2175' OR 'CGP 2175' OR 'CGP2175' OR 'H 93-26' OR 'H 93 26' OR 'H 9326' OR 'Metoprolol Tartrate' OR 'Seloken' OR 'Spesicor' OR 'Spesikor' OR 'Metoprolol Succinate' OR 'Metoprolol CR-XL' OR 'Metoprolol CR XL' OR 'Toprol-XL' OR 'Toprol XL' OR 'Beloc-Duriles' OR 'Beloc Duriles' OR 'Lopressor'
- Atenolol
  'Tenormine' OR 'Tenormin' OR 'ICI-66082' OR 'ICI 66082' OR 'ICI66082'
- Propranolol
  'Propanolol' OR 'Inderal' OR 'Avlocardyl' OR 'AY-20694' OR 'AY 20694' OR 'AY20694' OR 'Rexigen' OR 'Dexpropranolol' OR 'Dociton' OR 'Obsidan' OR 'Obzidan' OR 'Propranolol Hydrochloride' OR 'Hydrochloride, Propranolol' OR 'Anaprilin' OR 'Anapriline' OR 'Betadren'
- Nadolol
  'SQ-11725' OR 'SQ 11725' OR 'SQ11725' OR 'Corgard' OR 'Solgol'

#2 Long QT syndrome

'Electrocardiogram QT Prolonged' OR 'Romano-Ward Syndrome' OR 'Romano Ward Syndrome' OR 'Syndrome, Romano-Ward' OR 'Ventricular Fibrillation with Prolonged QT Interval' OR 'Long QT Syndrome Type 1' OR 'Long QT Syndrome 1' OR 'Ward-Romano Syndrome' OR 'Syndrome, Ward-Romano' OR 'Ward Romano Syndrome'

#3 RCT

'Randomized Controlled Trial' OR 'Controlled Clinical Trial' OR 'randomized':ti,ab OR 'placebo':ti,ab OR 'randomly':ti,ab OR 'trial':ti,ab OR 'groups':ti,ab OR 'drug therapy':lnk

#1 and # 2 and #3

Total number: PUBMED 37 + EMBASE 159 + CENTRAL 16 + OVID 187 = 399
Additional records identified through manual search: 3
Duplication: 77
Record excluded based on title, abstract, and detailed evaluation: 309
Final total number: 16
